# Diagnostic significance of urinary long non-coding PCA3 RNA in prostate cancer

**DOI:** 10.18632/oncotarget.17272

**Published:** 2017-04-20

**Authors:** Tao Wang, Xiangyun Qu, Jiajia Jiang, Peng Gao, Dingding Zhao, Xueqi Lian, Xiaohua Li

**Affiliations:** ^1^ Key Laboratory of PreMed Precision Medicine, Soochow University, Suzhou, 215121, China; ^2^ Jiangsu Provincial Key Laboratory of Molecular Biology and Translational Medicine of Malignant Tumor, Suzhou, 215123, China; ^3^ Affiliated AoYang Hospital of Jiangsu University, Zhangjiagang, Suzhou, 215600, China; ^4^ Nanjing Medical University, Nanjing, 211166, China

**Keywords:** prostate cancer antigen 3, prostate-specific antigen, prostate cancer, diagnostic signature, cell-free

## Abstract

Prostate cancer antigen 3(PCA3) is a long non-coding RNA, which was found increased expression in CaP patients than healthy individual. In this study, the individual nucleic acid of PCA3 and PSA was recombinant expressed as a reference reagent, and a quantitative RT-PCR with TaqMan assay was developed to examine the copies of PCA3 and PSA gene in urine. The results showed that the area under the receiver operating characteristic curve (AUROC) was 0.717, 0.444 and 0.916 for the number of PCA3 copy, PSA copy and for the score of PCA3/PSA RNA, respectively. Additionally, the AUROC for serum tPSA was 0.674 with a low specificity of 12.07%. Finally, the algorithm of PCA3 RNA *versus* PSA RNA was evaluated and corroborated as CaP biomarker by conducting a multicentric clinical trial. This study not only validated the developed technique of qRT-PCR with TaqMan assay for examination of urinary PCA3 and PSA RNA, it also demonstrated that the score of the PCA/PSA RNA was a reliable signature for CaP diagnosis.

## INTRODUCTION

Prostate cancer (CaP), most of adenocarcinoma, is a male-specific malignant disease and leads to high mortality in developed countries. Early stage of CaP is curable. However there is no effective treatment currently available for the later stage of disease. Clinically, the risk-stratification is based on the following three variables: total serum prostate-specific antigen (tPSA) level, the grade of prostate cancer (Gleason grading system) and the clinical stage of cancer (based on physical examination and imaging studies). Currently, testing tPSA is used for screening the malignancy of prostate epithelium, and the patient's biopsy analysis is still served as a golden standard for the CaP diagnosis in clinical practice.

PSA, a member of the kallikrein-related peptidase family, is encoded by the *KLK3* gene in human and produced / secreted out by the epithelial cells of the prostate gland. The transcription of KLK3 gene was documented to be regulated by androgen receptor (AR) [1, 2]. Thus, the activity alteration of AR affects KLK3 transcription and the expression of PSA. Abnormal expression and activation of AR, a common phenomena of malignant changes of prostate epithelium, was believed to be a driving force of CaP progression (see the review) [3]. Therefore, PSA level in blood and other body fluid including prostatic biopsy and urine should be an ideal biomarker for monitoring malignant status of prostate epithelial cell theoretically. Testing serum tPSA is currently applied as a routine procedure for initial screening of CaP. When a high level (≥4 ng /ml) of tPSA is detected, it signifies that a prostatic biopsy and histopathological analysis is necessary to distinguish possible malignant changes from other diseases in the prostate epithelium. However, increasing expression of PSA was also observed in prostatitis and benign prostatic hyperplasia (BPH), which resulted in a significant higher negative biopsy with unnecessary invasive clinical operation. Thus, this false-positive tPSA test-led both “overdiagnosis” and subsequent “overtreatment” brought about the high costs for society and painful experiences of invasive biopsy operations for patients [4, 5].

An early study found that expression of prostate cancer antigen 3 (PCA3) gene was increased in CaP tissue with significantly lower level in adjacent noncancerous tissue and no detectable level in normal tissue and other organelles [6, 7]. This characteristic of PCA3 makes it an ideal biomarker for malignant surveillance of prostatic epithelium [8]. PCA3 gene is located on the long arm of chromosome 9 with 23 kb long of nucleic acid and four exons. Due to various of the termination codons, PCA3 RNA was not translated into protein and was defined as a long non-coding RNA [7]. Testing PCA3 RNA in body fluid and designing a felicitous PCA3-based algorithm in combination with other molecules are expected to reflect malignant changes of the prostate epithelium. Below, we report the results of our attempts at: 1) developing and validating a method of qRT-PCR with TaqMan assay to quantify the copies of PCA3 and PSA genes in urine; 2) evaluating the diagnostic value of the algorithm of PCA3 *versus* PSA RNA in a multicentric clinical application of prostate oncology.

## RESULTS

### PCA3 expression in prostatic tissue

**Figure 1:**
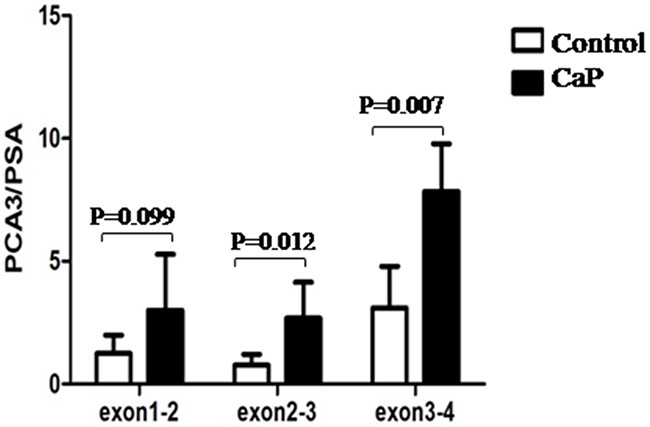
Amplification of PCA3 fragments The prostatic tissue from individual was collected and snap frozen immediately in -80°C. Total RNA was isolated and subjected to RT-PCR assay. Data was represented as a ratio of PCA3 RNA *versus* PSA RNA.

There are four exons allocated in the PCA3 gene. To select an appropriate exon of PCA3 nucleic acid for amplification, prostate tissues from five of control individuals and eight of patients with CaP were collected, and total RNA of each specimen was isolated and subjected to qRT-PCR analysis for PCA3 and PSA expression. The data showed that the pair of primers for amplifying exon 3-4 fragment of PCA3 generated the highest score of the ratio in either cancerous prostate tissue or control tissue when compared with using other two pairs of primers to amplify two of other fragments accordingly, and it also showed significant difference between the CaP group and the control group (p=0.007, Figure 1). Then this pair of primers was used with a special designed probe in the later procedure of qRT-PCR with TaqMan assay for quantification of the urine PCA3 copy in this study.

### Development of qRT-PCR with TaqMan assay for urinary PCA3 and PSA RNA examination

**Figure 2:**
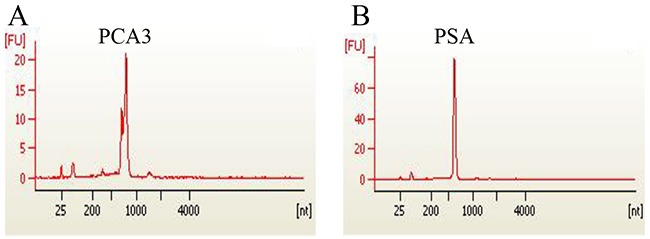
Recombinant expression of PCA 3 and PSA genes Gene clone, plasmid construction and transcription *in vitro* were conducted to obtain individual PCA3 RNA and PSA RNA. The quality of isolated RNAs of PCA3(A) and PSA(B) were analyzed and confirmed using Agilent 2100 Bioanalyzer (USA).

To generate ribonucleic acid standard for quantification of urine PCA3 and PSA by qRT-PCR, both genes of PCA3 and PSA were recombinant expressed, and RNA were isolated *in vitro* and purified, respectively. The ratio of OD260 over OD280 of isolated RNA were all located between the range of 1.8~2.0 with the clear separation of 28s and 18s fragments *via* running agarose-gel electrophoresis (data not shown). Analyzing by using Agilent 2100 Bioanalyzer, a single peak of RNA product in the location of 680 nt for PCA3 and in the location of 597 nt for PSA were also showed, respectively (Figure 2). The RNA concentration was presented as copies/μL using the following convert formula: copies/μL= (6.02×10^23^) × (RNA concentration of ng/μL×10^-9^sample) / (RNA length×340).

**Figure 3:**
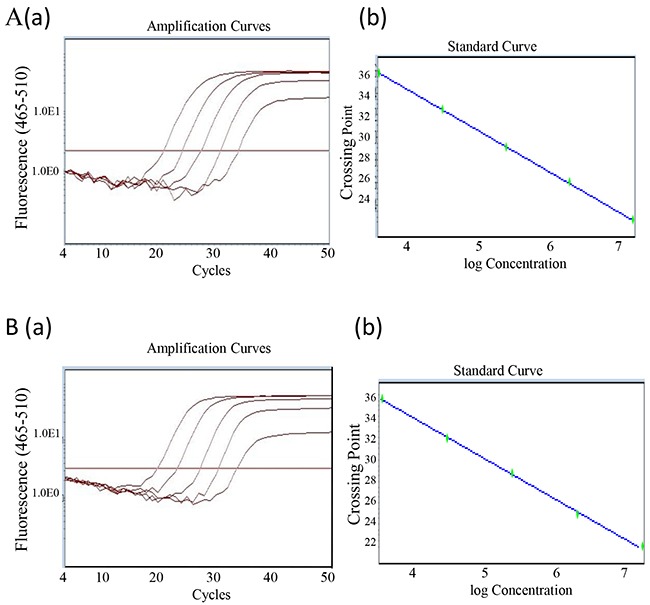
Generation of PCA3 and PSA standard curve **(A)** Recombinant expressed PCA3 RNA was serially diluted by ten-fold from 1.25 × 10^7^ to 1.25x 10^3^ (copy/ml). A qRT-PCR with TaqMan assay was conducted. The amplification curve **(a)** and the standard curve **(b)** were generated and presented. The PCA3 amplification efficiency was101.5%, the slope was calculated as -3.286, R^2^ =1.00. **(B)** The similar procedure described as above was performed for PSA. PSA amplification curve **(a)** and the standard curve **(b)** were also generated and presented. The amplification efficiency was 113.6%, the slope was -3.034, and R^2^=1.

To generate the standard amplification curve, the RNA was diluted by 10-fold and subjected to qRT-PCR with TaqMan assay. The data showed that there was a significant correlation between the RNA concentration and the number of amplification cycles for both PCA3 and PSA examination (r near to one). The linear range allocated from 1.25 × 103 to 1.25 × 107 for PCA3, and 6.25 × 10^3^ to 6.25 × 10^7^ for PSA (Figure 3A, 3B). Thus, the individual RNA, as reference reagent, was validated for quantificational analysis of the PCA3 and PSA RNA in urine.

### Algorithm of urinary PCA3 and PSA RNA in prostate cancer diagnosis

**Figure 4:**
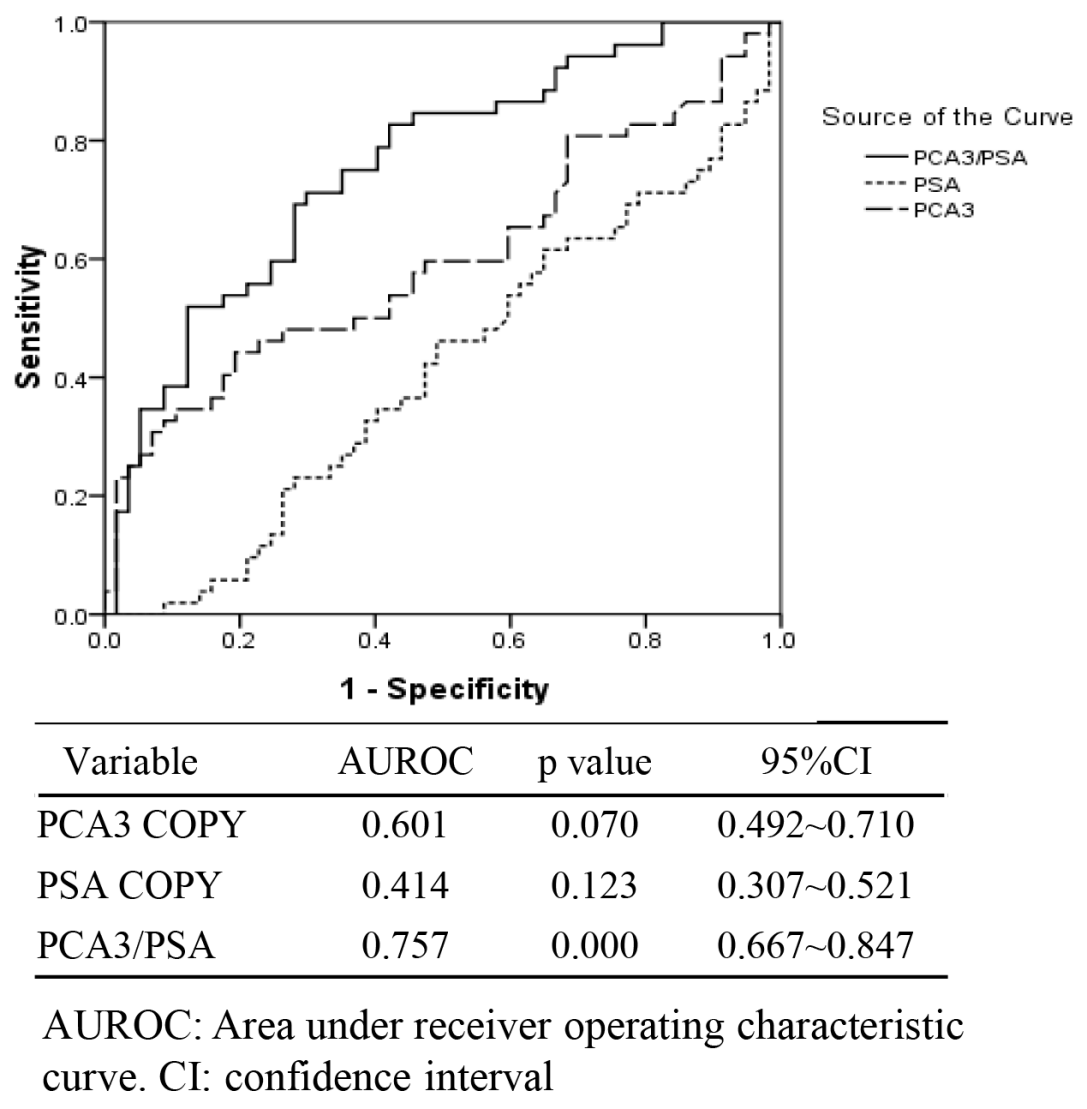
The receiver operating characteristic curve (ROC) for the copy of urinary PCA3 gene, the copy of urinary PSA gene and the score of urinary PCA3 / PSA RNA ratio (PCA3/PSA) in intended use of specimens (n=109)

To evaluate the value of PCA3 and PSA RNA level in urine for CaP predication or diagnosis, both urine specimen and clinical data from total 109 individuals of CaP patients and noncancerous control were collected. All the CaP patients of adenocarcinoma were proved by biopsy analysis. The urine total RNA was isolated and subjected to qRT-PCR with TaqMan assay for PCA3 and PSA expression. The obtained data was then analyzed statistically by running SPSS program and the ROC was plotted. Compared with the single copy number of PCA3 or PSA gene expression, the AUROC of PCA3/PSA RNA ratio was the biggest among the three groups. The score of PCA3/PSA ratio, other than their individual copy number, showed a significant correlation with the clinical diagnosis of CaP (p<0.001) (Figure 4 with the under table). Similar results were also obtained by using additional logistic regression analysis of likelihood ratio data (Table 1). Further statistics analysis indicated that using the copy number of PCA3 and the score of PCA3/PSA RNA ratio, but not PSA copy number, showed significant diagnostic value in CaP disease (p=0.009 for PCA3 copy, p=0.008 for PCA3/PSA, p=0.035 for PSA copy) (Table 2). The data demonstrated that the algorithm of urine PCA3/PSA RNA, with overall highest sensitivity and specificity (AUROC=0.757), could be a useful diagnostic signature in prostate oncology.

**Table 1:** Logistic regression analysis of likelihood ratio

	χ^2^	p
**PCA3 COPY**	5.243	0.155
**PSA COPY**	4.887	0.180
**PCA3/PSA**	39.033	0.000

**Table 2:** The statistic analysis of urine PCA3 RNA and PSA RNA in implement of CaP diagnosis

Group	N	PCA3 COPY			PSA COPY			PCA3 / PSA		
		Mean	SD	p	Mean	SD	p	Mean	SD	p
**Positive**	52	25560.43	42505.53		159358.46	179751.25		181.73	253.81	
**Negative**	57	8495.90	21787.98		339761.65	585921.87		65.97	190.90	
**Total**	109	16636.78	34243.85	0.009	253697.74	448845.00	0.035	121.20	229.55	0.008

### Evaluation of urinary PCA3/PSA RNA and serum tPSA in prostate cancer diagnosis

**Figure 5:**
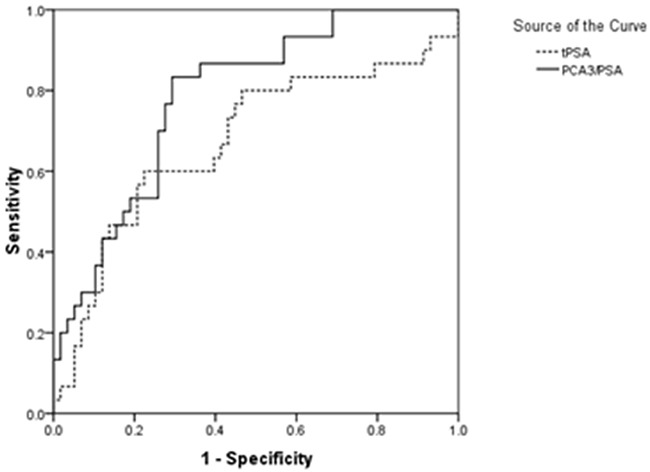
The receiver operating characteristic curve (ROC) for the score of urinary PCA3/PSA RNA ratio and serum tPSA in intended use of specimens (n=88)

Currently, examination of serum tPSA is the most commonly used procedure for prostate cancer screening. To distinguish the value of urine PCA3 *versus* PSA RNA in CaP diagnosis comparing with PSA test, both the urine and blood plasma were collected at the same time from individuals. A cohort of 88 individuals was involved in the study including patients with positive biopsy of CaP (n=30) and negative biopsy as control (n=58). The ROC curves of PCA3/PSA and tPSA were plotted out (Figure 5, Table 3). The data showed that the AUROC was 0.784 for PCA3/PSA RNA, but it was 0.674 for serum tPSA. Statistics analyses by chi-square test showed that, as prospectively, there is significant correlation between frequency of abnormal higher score of urine PCA3/PSA RNA (≥cutoff 40.38, Pearson Chi-square= 15.935, Kappa=0.421, P<0.05), rather than the frequency of higher tPSA (≥4.00 ng/ml, Pearson Chi-square=0.029, Kappa= -0.009, P>0.05), and the number of diagnostic CaP patients with positive clinical biopsy. Thus, with an acceptable sensitivity of 70% and specificity of 74.14%, our algorithm of using the ratio of urine PCA3/PSA RNA showed more accurate in prostatic malignancy examination and better as a tool than the currently used biomarker of serum tPSA (Table 3).

**Table 3:** The analysis of total serum PSA protein and urine ratio of PCA3/PSA comparing with prostatic biopsy results

		Serum tPSA		PCA3 / PSA	
		+	-	+	-
**Prostatic biopsy**	+	26	4	21	9
	-	51	7	15	43
**Sensitivity**		0.8667		0.7000	
**Specificity**		0.1207		0.7414	
**Coincidence rate**		0.3750		0.7273	

### Clinical evaluation of urinary PCA 3/PSA RNA in CaP diagnosis and prognosis

To further investigate the significance of urinary PCA3 *versus* PSA RNA in application of clinical CaP diagnosis, a larger scale of total 594 clinical patients were recruited. Both their urine and prostate biopsy were collected when they came to the clinic for their concern of prostatic malignancy at the first time. The PCA3 and PSA RNA in the urine were tested along with the clinical prostatic biopsy analysis. The results showed that there was significant correlation between the frequency of abnormal score of urine PCA3/PSA RNA and the number of clinical diagnostic CaP patients *via* histological biopsy analysis(p<0.001). The testing showed the sensitivity of 66.27% and the specificity of 80.94% (Table 4), which were similar with above described results.

**Table 4:** The analysis of urine RNA level of PCA3/PSA in clinical prostate cancer diagnosis

		Clinical diagnosis		
		CaP	Control	total
**Urine PCA3 /PSA RNA**	+	112	81	193
	-	57	344	401
	total	169	425	594
**Sensitivity (%)**		0.662721893		
**Specificity (%)**		0.809411765		

To study significant of the score of PCA3/PSA RNA in monitoring the cancer progress and prognosis, the data of Gleason score for a cohort of 20 CaP patients was extracted from their medical records. No correlation was found between Gleason score and the score of urinary PCA3 / PSA RNA (p=0.536, Pearson correlation coefficient = -0.143) (detail data not shown). This result indicated that testing urinary PCA3 and PSA RNA and conducting the algorithm of PCA3 / PSA RNA may have its’ limitation in monitoring disease progress.

## DISCUSSION

Currently, screening for CaP is conducted through the measurement of serum PSA. The digital rectal examination followed by a histological analysis of prostate biopsy is still served as golden standard for CaP diagnosis based on the guideline of NICE 2008 [9]. However, tPSA level was reported to be impacted by multiple factors, which interfered with the interpretation of tPSA in CaP diagnosis. Thus, exploring novel molecule and/or algorithm as risk predictor or diagnostic signature are desirable to improve the diagnostic efficiency for CaP.

In studying of three groups of tested individual, we found that there was no significant correlation between high serum tPSA and positive biopsy results (Table 3, Figure 5, p<0.05) and cannot predicate the risk of CaP accurately. However, the score of urinary PCA3 RNA *versus* PSA RNA showed a more favorable benefit than the single copy of PCA3 RNA, PSA RNA and serum tPSA for diagnosis of CaP. Meanwhile, our method achieved very satisfactory sensitivity and specificity in test [10, 11]. Notably, the urine sample was collected after the DRE operation and without conducting prostatic massage which is different from the reported method [12, 13]. It could avoid the extra procedure-induced none specific release of targeted molecules and reserve the test's specific. In addition, we applied preservative reagent STR for urine collection and storage without affecting the sample quality for the assay [12]. In this way, it is convenient for the sample to be handled for transferring and for laboratory management.

Since the claim is that “DD3 is one of the most prostate cancer-specific genes yet described” [7], testing PCA3 level in variants clinical specimens including blood, urine, ejaculate, and other body fluid was sequentially proposed and clinically conducted [14–16]. For example, examining PCA3 and PSA expression in peripheral blood, Vaananen et al reported that PCA3 nucleic acid was amplified positively from two out of nine patients with metastasis phenotypes, eight samples were PSA positive, no PCA3 expression in healthy individuals from a cohort of 91 tested prostatic cancer/disorder patients and healthy controls [16]. The data suggested that, when compared with examination of PSA, testing blood PCA3 potentiated high specificity, rather than sensitivity for CaP diagnosis. In addition, Fonseca Coelho et al reported that both the number of PCA3 copies alone and the ratio of PCA3/PSA RNA in urine were significantly higher in CaP group than in the BPH group. Neither PSA gene expression nor serum tPSA was found significant difference between these two groups [13]. It was also reported that examination of PCA3 owed the diagnostic value for CaP, typically for the patients with disease grade of Gleason score ≥7 [17, 18].

Over the past decade, examination of PCA3 gene expression by RT-PCR was extensively studied to test its predictive and diagnostic value for prostate malignant disorder [19, 20]. The first detection kit, named as Progensa PCA3 assay, has undergone evaluation in clinical laboratories [19]. However, the clinical advantage of PCA3 RNA detection is still in debate due to existing variable results in the diagnosis of CaP [13, 21]. Obviously, as a procedure of no invasive operation, testing urinary PCA3 RNA possesses its advantage in clinical prostate oncology. In addition, the attempts have been continually made to explore a novel biomarker for CaP screening and diagnosis. Nucleotide molecules including none coding RNAs (e.g. PCAT1, SCHLAP1, PCGEM1, PRNCR1) were discovered and showed promising outcomes for this purpose [12, 22]. The algorithm based on the analysis of ERG, PCA3 and SPDEF showed its advantages in discriminating the high-grade cancer (≥GS7) from low-grade malignancy (GS6) and benign disease than tPSA in CaP screening [23]. HOXC6 and DLX1 mRNA in urine were also reported as an ideal predictor for malignant prostatic epithelial progress [24]. Obviously, discovering a novel biomarker and developing a feasible methodology for CaP screening, diagnosis, and for monitoring the disease progress are still desirable in prostatic oncology.

## MATERIALS AND METHODS

### Study design and specimen collection

This study was conducted by strictly following the protocol approved by our Research Ethics Committee, the Affiliated Aoyang Hospital of Jiangsu University. The informed consent was also obtained from all subjects. Prostate tissue specimen was collected from the following participating hospitals: Department of Oncology, Peking Union Medical College Hospital (Beijing, China). The Fifth People's Hospital of Shanghai, Fudan University (Shanghai, China). Beijing Cancer Hospital (Beijing, China). Peking University First Hospital (Beijing, China). Beijing Chao-Yang Hospital (Beijing, China). Shanghai Tongji Hospital (Shanghai, China). The First Affiliated Hospital of Soochow University (Soochow, China) and Affiliated AoYang Hospital of Jiangsu University (Suzhou, China). The study included clinical specimen from prostate adenocarcinoma patients and the control group of healthy individuals without hyperplasia. The median age (range) was 66(45~90) years old for CaP patients and 65(45~92) years old for control individuals. The patients with adenocarcinoma were clinically approved according to the Diagnosis Criteria for Prostate Cancer (WS/T 336-2001, China) and the guideline of NICE 2008 [9]. The cancer malignant grade was evaluated by Gleason Score given according to the modified evaluation system based on 2005 consensus conference [25]. The fresh tissue specimen collected from the prostate surgery operation of five healthy individuals without hyperplasia and eight CaP patients were snap frozen in -80°C.

When collecting the urine specimen, the patients or control healthy individuals took 500 ml of water firstly, and then underwent DRE (digital rectal exam) followed by urine collection without massage. The total amount of 5 ml mixed-urine was collected in a sterilized container which was prefilled with equal amount of 5 ml specially designed Specimen Transport Reagent (STR, 50 mM LiDS, 10 mM NaH_2_PO_4_, 10 mM Na_2_HPO_4_, 1 mM EDTA, 1 mM EGTA, pH 6.7) for RNA isolation. The RNA in collected urine samples is stable when it was stored in 4°C for three days, or -20°C for six months, and the urine sample is suitable for the analysis.

### Gene clone, plasmid construction and expression [26]

To obtain the reference RNA for generation of standard cure for quantification of gene expression, the whole cDNA of PCA3 and PSA containing T7 promoter sequence and two restrictive enzyme sites of BamH and XbaI were amplified by polymerase chain reaction (PCR) from prostate epithelial tissue, respectively. The primer sequence for PCA3 was GGATCCTAATACGACTCACTATAGGGACAGAGGGGAGATTTG and TCTAGATGAGGGTTAGAAATATGAAATGATT; for PSA was GGATCCTAATACGACTCACTATAGGGAGACCAAGTTCATGCT and TCTAGA GTGCTTCATGGACAGTGTCCAGCACA. All the nucleotide sequences were synthesized at Thermo Fisher Scientific (Shanghai, China). The individual product of amplified nucleic acid was then cloned into pGM-T vector using pGM-T ligation kit (TIANGEN Biotech Co., Ltd. Beijing). The ligation product was introduced into DH5α cell, and the plasmid DNA in growing E. coli clone was subsequently sequenced. The verified plasmid sequence in clones was purified for gene expression by utilizing the T7 RiboMAX^TM^ Express Large Scale RNA Production (Promega, USA). Total RNA was isolated by using RNeasy mini kit (QIAGEN, Germany). The products of PCA3 RNA and PSA RNA were analyzed by using Agilent 2100 Bioanalyzer (Agilent Technologies, USA). The individual RNA was used as a reference for quantification analysis along with PCR amplification simultaneously.

### Quantitative reverse transcription-polymerase chain reaction (qRT-PCR) [26, 27]

Magnetic beads preparation: The magnetic beads were stored in refrigerator and were washed for three times with 20-fold volume of Target Capture Reagent (TCR, 150 mM HEPES, 0.5 M LiCl, 450 mM LiOH, 100 mM EDTA, pH 6.4). The capture sequence for PCA3 was ACAGGGCGAGGCUCAUTTTA(dA)_30_, and for PSA was CAAUAGGGAGUUGAUAGGTTTA(dA)_30_. The individual capture sequence was added at final concentration of 10 M and mixed at room temperature for 10 min. The supernatant was then removed and the beads were re-suspended in 20-fold volume of TCR solution for urinary RNA isolation.

RNA isolation: To isolate the urinary RNA, 800 μl of collected urine specimen plus 200 μL capture magnetic beads were mixed and incubated in 62°C water bath for 10 min and then in room temperature for 40 min. The mixture was pulse centrifuged and the supernatant was removed. The pellet of beads were then washed once with wash solution (250 mM HEPES, 200 mM NaCl, 6.5 mM NaOH, 1 mM EDTA, 0.5% ethanol, 0.02% Methylparaben, 0.01% Propyl 4-hydroxybenzoate, 1% SDS, pH 7.5), and were re-suspended in 30 μL of pre-warmed (75°C) elution buffer (5 mM Tris-Hcl, pH7.5). After mixed and incubated in 75°C water bath for 2 min, the beads were precipitated by centrifuging, and sample supernatant was harvested for gene expression analysis. In addition, total tissue RNA was isolated using RNeasy mini Kit (Qiagen, Germany). The RNA concentration and quality was analyzed using NanoDrop 2000 spectrophotometer as described above (Thermo, USA).

Quantification of *PCA3* and *PSA* gene expression in tissue: One-Step absolute quantification real-time PCR was developed to determine the individual gene expression of PCA3 (NR_132312.1) and PSA (Human Kallikrein, NM_001030048.1). When conducting the test, the laboratory technician was muddled with the subject's clinical status by randomizing the order of the specimen. The primer sequences for amplifying PCA3 exon1-2(cover 98~198 of gene NR_132312.1) were GGGAAGGACCTGATGATACAG and AGGAGACTCACAGCGGACTA; for amplifying PCA3 exon2-3(cover 219~310 of NR_132312.1), ACCATCGACGGCACTTTC and GTCAGCAGCCTTTCTTATTTCT; for amplifying PCA3 exon3-4 (cover 440~507 of NR_132312.1), ACACAGGAAGCACAAAAGG and GATGACCCAAGATGGCGGC; The primer sequence for amplifying PSA (535~688 of NM_001030048.1) was CCTGCTCGGGTGATTCTG and GCCACGATGGTGTCCTTGAT. The One-Step qRT-PCR was conducted in total 50 μl of reaction solution containing 1 x PCR buffer, 2.5 mM MgCl_2_, 0.25 mM dNTP, 2 units of reverse transcriptase, 2 units of RNase inhibitor ( all from TAKARA), 0.25 mM of each primer, 0.2 mM probe, 1 unit of UDG (NEB), 4 units of HS Taq DNA polymerase (Roche, USA), and 5 μL of RNA template from tissue or 24 μL of urine specimen. The analysis was incubated at 37°C, 5 min for UDG-mediated decontamination of DNA, 50°C, 15 min for reverse transcription, then 94°C, 5 min, followed by 94°C, 10 sec and 60°C, 30 sec for total 50 cycles, ended at 50°C, 30 sec.

Quantification of PCA3 and PSA RNA in urine: To exam the urinary PCA3 and PSA RNA levels, a qRT-PCR with TagMan assay was developed by our team, and a standard curve for the each target gene was generated simultaneously by using above recombinant expressed nucleic acid of *PSA or PCA3* as a reference reagent. Each concentration point for standard curve generation was analyzed in triplicate, while each sample was analyzed in multiple replicates. The primers for amplification of both PCA3 (amplifying exon3-4) and PSA were listed above. The probe sequence of PCA3 (target 461~486 of NR_132312.1) was 5’-FAM-GCACAGAGATCCCTGGGAGAAATGCC-MGB 3’; of PSA (target 554~579 of NM_001030048.1) was 5’-FAM-GGGCCCACTTGTCTGTAATGGTGTGC-MGB 3’. The RNA level of each gene was quantified by qRT-PCR. The score was designed as the ratio of PCA3/PSA RNA calculated as (the number of PCA3 copy / the number of PSA copy) x1000, and the cutoff score was pre-determined as 40.38 according to the principle of previous study [13, 28]. If the score is bigger than the cutoff value, the result is considered as positive. Otherwise, the result is negative.

### Total serum prostate-specific antigen (tPSA)

The blood plasma from each individual was obtained and the total PSA level was evaluated by using Total Prostate-Specific Antigen Quantitative Detection kit (Chemiluminescent Immunoassay, Kehua Bio-Engineering co., Ltd, Shanghai) in the clinic laboratory. The test was conducted in a blind manner described above. If the level of serum tPSA is greater than 4.00 ng/ml, the results are considered suspicious, which then warrants a prostatic biopsy examination and histological analysis [29]. Less than 4.00 ng/ml of serum tPSA is considered as negative, and the patient does not need for any further clinical care.

### Statistical analyses

The Statistical Package for Social Sciences (SPSS, Version 19.0) was used for statistic analysis. The receiver operating characteristic (ROC) curve was plotted to evaluate the sensitivity and specificity of the variables biomarker used in the diagnosis of prostate abnormalities. The one –way analysis of variance (ANOV) and chi-square test were applied to compare the categorical variable frequencies and significant differences. Logistic regression analysis of likelihood ratio for the data was also conducted to double confirm the analysis results. The difference was considered statistically significant at a p-value <0.05.

## Abbreviations

PCA3: prostate cancer antigen 3
tPSA: total serum prostate-specific antigen
CaP: prostate cancer
qTR-PCR: Quantitative reverse transcription-polymerase chain reaction
AUROC: areas under the receiver operating characteristic curve
DRE: digital rectal exam
